# *Propionibacterium acnes* and Acne Vulgaris: New Insights from the Integration of Population Genetic, Multi-Omic, Biochemical and Host-Microbe Studies

**DOI:** 10.3390/microorganisms7050128

**Published:** 2019-05-13

**Authors:** Joseph McLaughlin, Steven Watterson, Alison M. Layton, Anthony J. Bjourson, Emma Barnard, Andrew McDowell

**Affiliations:** 1 Northern Ireland Centre for Stratified Medicine, School of Biomedical Sciences, Ulster University, Londonderry BT47 6SB, UK; McLaughlin-J35@ulster.ac.uk (J.M.); s.watterson@ulster.ac.uk (S.W.); aj.bjourson@ulster.ac.uk (A.J.B.); 2 Department of Dermatology, Harrogate and District NHS Foundation Trust, Harrogate HG2 7SX, UK; laytona@btinternet.com; 3 School of Biological Sciences, Queen’s University, Belfast BT9 7AE, UK; e.barnard@qub.ac.uk

**Keywords:** *Propionibacterium acnes*, *Cutibacterium*, phylogroups, MLST, clonal complex, sequence types, multi-omic analyses, virulence factors, host-microbe interactions, novel therapeutics, vaccination

## Abstract

The anaerobic bacterium *Propionibacterium acnes* is believed to play an important role in the pathophysiology of the common skin disease acne vulgaris. Over the last 10 years our understanding of the taxonomic and intraspecies diversity of this bacterium has increased tremendously, and with it the realisation that particular strains are associated with skin health while others appear related to disease. This extensive review will cover our current knowledge regarding the association of *P. acnes* phylogroups, clonal complexes and sequence types with acne vulgaris based on multilocus sequence typing of isolates, and direct ribotyping of the *P. acnes* strain population in skin microbiome samples based on 16S rDNA metagenomic data. We will also consider how multi-omic and biochemical studies have facilitated our understanding of *P. acnes* pathogenicity and interactions with the host, thus providing insights into why certain lineages appear to have a heightened capacity to contribute to acne vulgaris development, while others are positively associated with skin health. We conclude with a discussion of new therapeutic strategies that are currently under investigation for acne vulgaris, including vaccination, and consider the potential of these treatments to also perturb beneficial lineages of *P. acnes* on the skin.

## 1. Introduction

The human skin, which is the largest organ of the body, is composed of a variety of key microbial genera associated with skin health, including *Staphylococcus*, *Propionibacterium*, *Streptococcus*, *Corynebacterium* and *Malassezia* [1]. In particular, the Gram-positive anaerobic bacterium *Propionibacterium acnes* is a major resident of the normal human skin microbiota and dominates pilosebaceous units. Along with other well described cutaneous propionibacteria (*P. avidum, P. granulosum*), it is believed to play an important role in maintaining skin health via occupation of ecological niches that could be colonised by more pathogenic microbes; it produces short chain fatty acids, thiopeptides, bacteriocins and other molecules with inhibitory properties against such organisms [2,3,4]. *P. acnes* and *P. granulosum* are most abundant in the sebaceous gland-rich sites of the skin, which includes the face and upper trunk, although *P. acnes* can also be recovered from other body sites including the mouth, gastrointestinal tract and prostate, suggesting potential mutualistic effects that extend beyond the skin [5]. In contrast, *P. avidum* prefers colonisation of moist areas including sweat-rich axilla, nares, groin and rectum [6]. In keeping with a role in maintaining skin health, reduced abundance of propionibacteria have been observed on the skin of patients with the chronic skin diseases psoriasis and atopic dermatitis [7,8].

**Figure 1:**
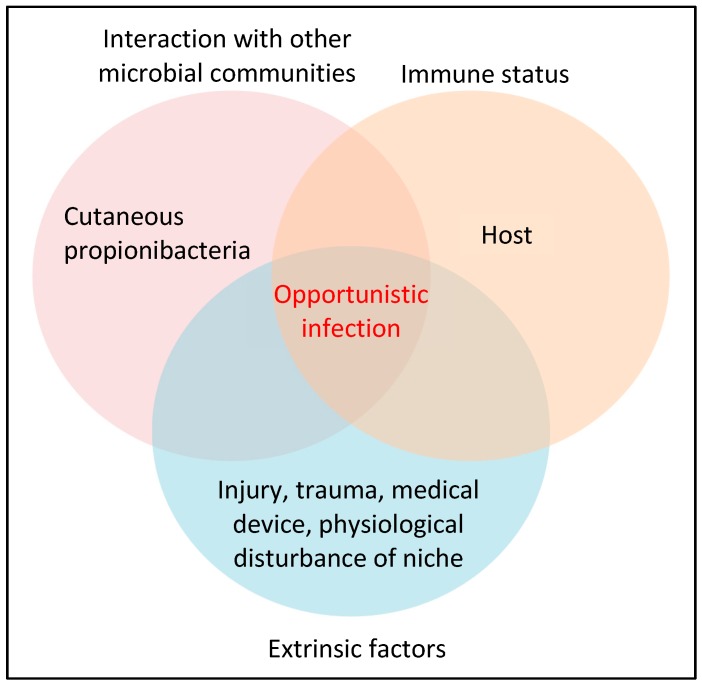
Key requirements for cutaneous propionibacteria, especially *P. acnes*, to cause an opportunistic infection.

While cutaneous propionibacteria help to maintain and support the natural microbial balance of the skin, they are not always beneficial and can cause disease given the correct set of conditions (Figure 1). Of the cutaneous propionibacteria, *P. acnes* appears the most frequent cause of opportunistic infection and is linked to a wide range of seemingly disparate conditions including the skin diseases acne vulgaris and progressive macular hypomelanosis (PMH), medical device-related and dental infections, sarcoidosis, cervical disc disease, prostate cancer and various soft tissue infections [9,10,11,12,13,14,15,16,17,18]. Indeed, over the last 20 years, there has been a growing recognition of the role of this pathobiont in human disease due, in part, to improved detection methods, such as adherence to strict anaerobic conditions while processing clinical samples, as well as extended anaerobic culture incubation times (14 days).

The purpose of this review is to re-focus on the role of *P. acnes* in the skin condition acne vulgaris in light of new data emerging from population genetic, multi-omic and biochemical studies, as well as investigation of host-microbe interactions.

## 2. Taxonomy and Intraspecies Diversity of *Propionibacterium acnes*

**Figure 2:**
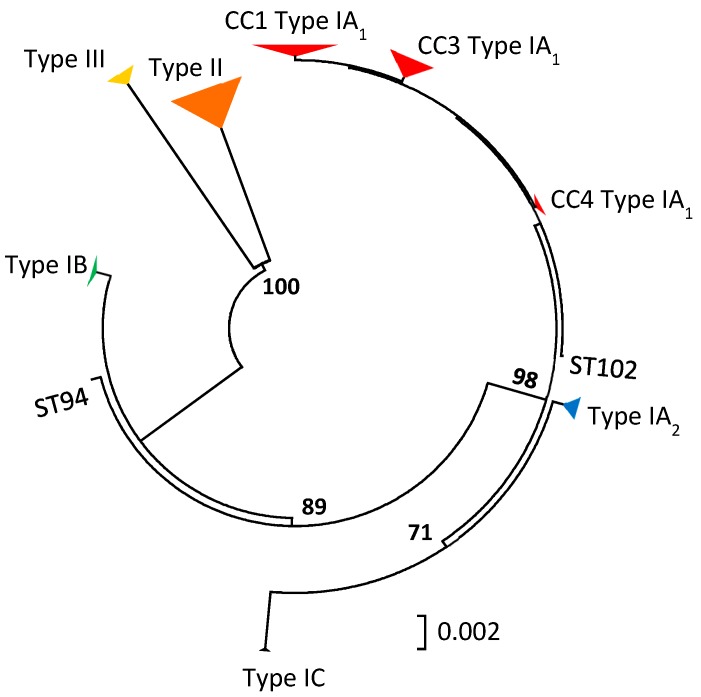
Minimum evolution phylogenetic tree of concatenated gene sequences (4253 bp) from all current STs in the MLST_8_ database. Bootstrapping statistics were performed using 500 data sets, and only bootstrap values ≥70% are shown. Clonal complexes (CC) are indicated.

The last ten years have seen a fast-moving shift in our understanding of the population genetic structure of *P. acnes* alongside changes to its taxonomic description [19,20,21,22,23,24,25]. As a result of extensive single, multi-locus and whole genome sequence analyses, the bacterium has been shown to have a clonal population structure and to comprise several distinct, major phylogenetic groups classified as types I, II and III, with the major type I clade being further divided into sub-clades known as types IA_1_, IA_2_, IB and IC (Figure 2). The developments in our understanding of the intraspecies phylogeny, as well as descriptions of the various clonal complexes (CC) and sequence types (STs) characteristic of the different phylogroups or sub-clades, have recently been reviewed in detail elsewhere, including current molecular methods to type the organism. As a result, we would refer the reader to this source for further information [26]. While two Multilocus Sequence Typing (MLST) schemes with similar levels of resolution have been described for *P. acnes*, for the purposes of this review we will restrict all discussion of CC and ST data to that from the MLST_8_ scheme [22,27], although both MLST_8_ and MLST_9_ [21] schemes have been used with similar frequency based on our detailed review of the literature.

As we will discuss later in this review, these distinct phylogroups vary with respect to a wide range of characteristics, including cellular morphology, aggregative and biochemical characteristics, inflammatory and immunogenic properties and production of virulence factors, which may also help to explain differences in their associations with health and disease [13,16,19,20,21,28,29,30,31,32]. Collectively, such studies have now led to the proposal that the major type I, II and III clades should be classified as distinct subspecies known as *P. acnes* subsp. *acnes*, *P. acnes* subsp. *defendens* and *P. acnes* subsp. *elongatum*, respectively [23,24]. Furthermore, a parallel proposal that the genus *Propionibacterium* should now be divided into four genera based on whole genome analysis and consideration of isolation source has also been made, with the cutaneous propionibacteria being reclassified within the new genus *Cutibacterium* [25]. This proposal has, however, proved controversial for a number of reasons [33], and also did not accommodate the subspecies proposals due to the overlapping timeline of the publications; very recently, the latter issue has been corrected [34]. For the purposes of this current review, and to prevent any confusion, we have decided to still use *Propionibacterium* (which it is still valid to do) [33] until the new genus name is broadly adopted by the wider medical microbiology community.

## 3. Acne Vulgaris

The chronic inflammatory and recurrent skin condition acne vulgaris, commonly referred to as acne, is a disease of the pilosebaceous unit (hair, hair follicle, erector pili muscle and sebaceous gland) and, strikingly, the eighth most prevalent disease globally, affecting approximately 10% of the world’s population [35]. The disease has a multifactorial aetiology and is triggered initially during adrenarche in susceptible individuals, and can be mild to very severe with respect to symptoms [36]. Furthermore, for a growing number of individuals, particularly females, the condition can continue, or occur for the first time, in adulthood [37]. Although not life-threatening, acne can have profound social and psychological effects, which are frequently more significant when symptoms are severe and scarring occurs.

In relation to acne pathogenesis, the perceived wisdom has always been that the condition develops within a follicle as a result of four main events: (1) androgen-induced hyperseborrhoea, (2) follicular hypercornification, (3) colonisation and proliferation of *P. acnes* and (4) stimulation of a local innate immune reaction [36,38]. These changes then cause a normal follicle or pore to evolve into an invisible subclinical precursor lesion known as a microcomedo, which then progresses to a non-inflammatory (open and closed comedones) and inflammatory lesion (papule, pustule or nodule). While comodogenesis was once thought to precede the inflammatory phase of lesion development, this view has now changed with evidence that inflammation may play a fundamental role in the development of microcomedone lesions, even before keratinocyte proliferation [39].

### 3.1. What is the Evidence that P. acnes is a Pathogenic Factor in Acne Development?

**Figure 3:**
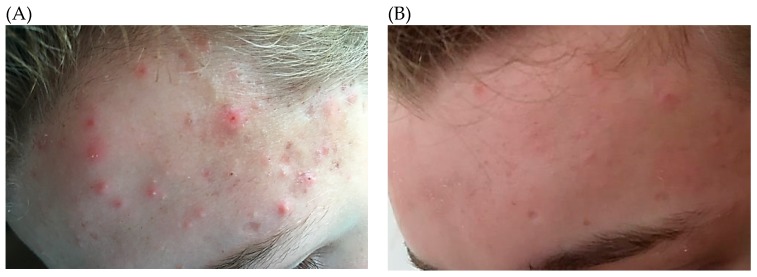
A 14-year-old adolescent boy who presented with moderate inflammatory and non-inflammatory acne lesions (**A**). After consultation and initial treatment for three months with oral minocycline (100 mg/d) and a topical antimicrobial gel, his condition was greatly improved (**B**).

As *P. acnes* forms part of the normal human microbiota, Koch’s postulates cannot be satisfied in relation to the establishment of a causal effect. Evidence for a role in the pathogenesis of inflammatory acne is therefore circumstantial, and primarily based on the observation that antimicrobial treatments are efficacious for the resolution of symptoms (Figure 3). Furthermore, non-response to antibiotic treatment is commonly associated with the presence of antibiotic-resistant strains, thus negating the argument that the effects of antibiotics are solely due to their anti-inflammatory activity [40]. In addition, it has been shown that patients with severe forms of acne have higher antibody titres to the bacterium compared to healthy controls [41].

Despite these observations, our understanding of how *P. acnes* contributes to the pathophysiology of acne still remains unclear and at times contradictory, even though it has been over a century since an association between the bacterium and acne lesions was highlighted [42]. In particular, skin surface concentrations of *Propionibacterium* do not differ between individuals with and without acne, and within follicles there does not appear to be any correlation between levels of the bacterium and the degree of inflammation [43]. Furthermore, some lesions are inflamed, yet show no evidence of viable bacterial colonization highlighting the importance of other factors in the initiation of the condition, such as altered sebum composition; notably, not all patients with acne display hyperseborrhoea [36].

In contrast, direct visualisation of *P. acnes* in skin biopsies by immunofluorescence microscopy (IFM) has revealed a statistically significant association of follicular *P. acnes* populations with acne versus controls. Furthermore, highly complex *P. acnes* macrocolonies/biofilms appear to be a more common phenomenon in acne than normal subjects and we can speculate that such structures could hinder sebum flow [44]. A further example of the changing views on the pathophysiology of acne can be seen with the recent suggestion that little evidence actually exists for follicular keratinocyte hyperproliferation in acne lesions versus autologous healthy hair follicles, and that alternate mechanisms may exist for infundibular hyperkeratinisation [45]. Collectively, such results serve to highlight the complicated and multifactorial nature of the disease process underlying acne and the challenges facing researchers, including fully elucidating the role played by *P. acnes*.

### 3.2. Inflammatory Responses in Acne: How does P. acnes Fit the Bill?

While the role of inflammation in the later stages of acne has been well accepted, it has become increasingly clear that the inflammatory process occurs much earlier in the evolution of the acne lesion and is not a secondary event [46]. In particular, formation of microcomedones is preceded by a lymphocytic infiltrate consisting of CD4+ T cells and CD68+ macrophages [46]; inflammation is, therefore, not initiated by neutrophils (as originally thought), which appear later in the process and lead to pustule formation [47]. Within comedones there is also a heightened production of biologically active IL-1α, which may be important in comedogenesis via IL-1α-induced hyperkeratinisation [48]. Increased expression of a wide range of cytokines and chemokines associated with the Th17 pathway has also been observed; these include IL-1β, IL-6 and TGF-β involved in Th17 lineage differentiation and proinflammatory TNF-α, IL-8, CSF2 and CCL20 [48]. IL-17A positive T cells and CD83 dendritic cells are also present in increased numbers [49].

The observation of inflamed lesions in the absence of bacteria would suggest that while *P. acnes* may not be a pre-requisite factor for initiation of inflammation it, at the very least, may exacerbate or intensify any response [50]. The strong immunostimulatory nature of *P. acnes* has been known for a long time, and the organism and its antigens have been shown in vitro to elicit both innate and adaptive inflammatory responses from key cell types found within the pilosebaceous unit including keratinocytes, sebocytes and monocytes [28,29,51] (see Section 3.7). The bacterium also produces lipases which facilitate breakdown of sebum (carbon source), a complex of different lipid types, into free short chain fatty acids (SCFAs) which are pro-inflammatory [52]. The capacity to elicit an inflammatory response from the host is mediated via pattern recognition receptors on antigen presenting cells, such as Toll-like receptors (TLR) 2 and 4 [53], and nucleotide oligomerization domain (NOD)-like receptors [54], as well as damage-associated molecular patterns [55]. Of particular note is the observation that *P. acnes* can activate the NOD-like receptor protein 3 (NLRP3)-inflammasome and the protease Caspase 1 of monocytes/macrophages and sebocytes, resulting in the production of IL-1β which is abundant in acne lesions [56,57]. Interestingly, inhibition of TLR2 can reduce the production of IL-1β, suggesting that it may also have a role to play in NLRP3 inflammasome activation. *P. acnes* also induces the expression of IL-17 from peripheral blood mononuclear cells (PBMCs) and promotes Th17 and Th1 response pathways [51,58,59]. In addition, *P. acnes* can also stimulate the release of antimicrobial peptides and degradative matrix metalloproteinases (MMPs) that further enhance inflammation [28,29,60].

### 3.3. Insights into the Role of P. acnes in Acne from Population Genetic Analyses

#### 3.3.1. Culture-Based Analysis

**Figure 4:**
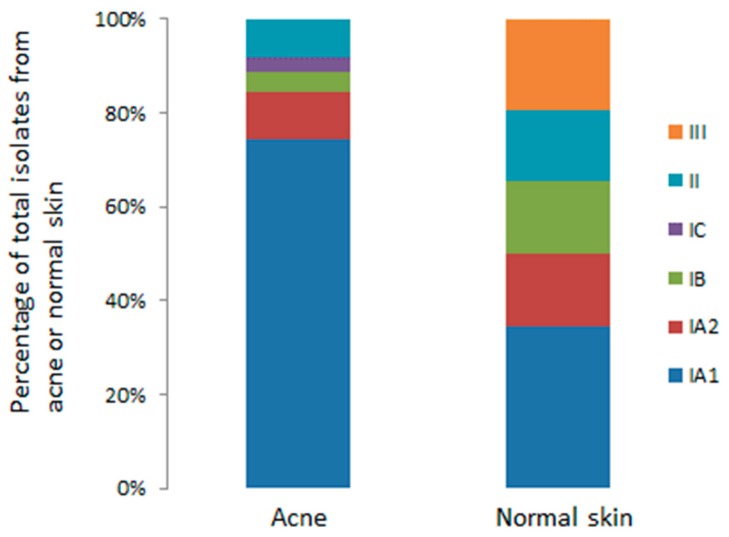
Association of *P. acnes* phylogroups with acneic and healthy skin. Data was analysed from the current MLST_8_ isolate database. Statistically significant differences (*p* < 0.001, Fisher’s exact test) were observed for type IA_1_ and type III distributions between acneic and healthy skin based on this isolate cohort (type IC numbers too small for statistical analysis).

Over the last decade, the development of various molecular typing methods for *P. acnes*, particularly MLST protocols validated by whole genome sequencing (WGS), has provided the opportunity to investigate the population genetic structure of the bacterium, and identify specific STs or lineages that may be associated with skin health and disease. Such studies have breathed new life into acne research and proved important in helping to address the issue of why *P. acnes* has a “Jekyll and Hyde” personality and functions as an important component of the normal skin microbiota, but also a potentially pathogenic factor in regard to acne and other diseases. Furthermore, identification of specific lineages associated with a disease state also ensures that the correct strains are utilised when trying to determine mechanisms of pathogenicity, especially in relation to host interactions. One issue that has complicated epidemiological investigations has been the use of various skin sampling techniques (swab, scrape, tape stripping, cyanoacrylate gel biopsy) which target different microbial populations (e.g., stratum corneum, infundibulum, lower hair follicle), and sample both acne and normal follicles [61]. Also, analysis of only a single isolate, while potentially identifying a dominant clonal lineage within a sample, may also miss other phylogroups present. Yet, despite these methodological issues, several independent culture-based studies have found that acne is strongly associated with strains from the type IA_1_ and IC clades, while those from the type IA_2_, IB, II and III phylogroups are much more frequently associated with skin health and other types of opportunistic infections and conditions [10,11,21,22,27,62]. Analysis of the *P. acnes* MLST_8_ isolate database (Available online: https://pubmlst.org/cacnes/), which contains information on hundreds of global isolates recovered from acneic and healthy skin (single and multiple) based on different sampling methods, reveals statistically significant enrichment of type IA_1_ strains with acne (face and back), while strains from comparable acne-prone areas of healthy skin are represented by a more balanced distribution of different phylogroups (Figure 4); with the large number of isolates analysed from multiple independent studies, this is indicative of a meaningful association.

**Figure 5:**
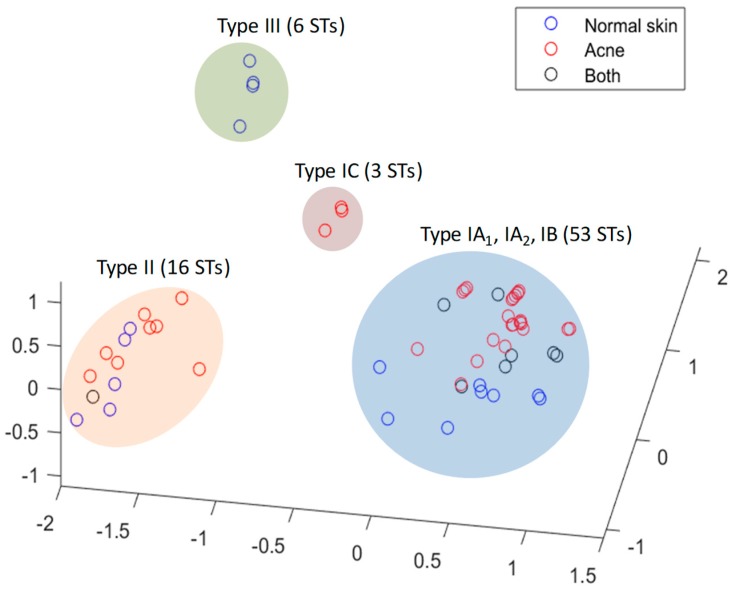
Principal component analysis (PCA) plot of *P. acnes* STs associated with acneic and healthy skin. Data is from the MLST_8_ isolate database. Using the presence or absence of an allele at each specific gene locus as a separate coordinate, each ST is represented here as a point in a 111 dimensional space. The PCA plot separates STs into four clusters representing types IA_1_/IA_2_/IB, type IC, type II and type III. Note: phylogroup III has never been associated with acne.

One other interesting observation from the MLST_8_ isolate database is that type III strains are the only phylogroup that have never been cultivated from acneic regions of the skin, at least to date, but have been found on normal skin (primarily the back) (Figure 5) and more recently have been linked to the skin condition PMH [10,11], thus suggesting they may not be true commensal bacteria. While isolates from other phylogroups have been recovered from acneic skin (IA_2_, IB, II) the rates of recovery are low; these phylogroups may, however, still prove clinically relevant to the condition in some instances thereby challenging the view of highly distinct acne- and non-acne-associated phylogroup lineages [63,64]. As we have proposed previously, the apparent loss of phylogroup diversity in acne and the dominance of type IA_1_ strains may reflect a dysbiotic shift within a follicle as microenvironmental changes lead to lesion formation [27]; alternatively, it could mean that certain individuals already lack *P. acnes* phylogroup diversity within their skin microbiota and are colonised with acneic type IA_1_ strains, thus predisposing them to acne in combination with other risk factors.

**Figure 6:**
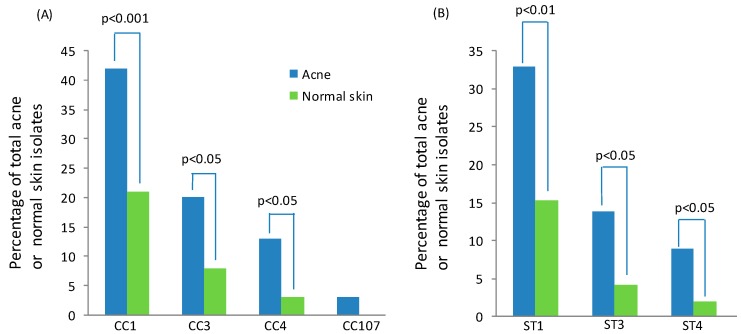
Association of type IA_1_ (CC1, CC3, CC4) and type IC (CC107) CCs (**A**), as well as ST1, ST3 and ST4 genotypes (**B**), with acneic and healthy skin. Data was analysed from the MLST_8_ isolate database. *p*-values were calculated using N-1 Chi squared test for independent proportions.

The type IA_1_ phylogroup is composed of three major CCs, known as CC1, CC3 and CC4, all of which contain STs that are associated or enriched in acneic versus healthy skin (Figure 6A). Within these CCs, the founding genotypes ST1, ST3 and ST4 appear particularly dominant lineages associated with acne (Figure 6B), but are also part of the normal skin microbiota as well as globally disseminated. The type IC CC, known as CC107, is also acne associated (Figure 6A), but the number of isolates recovered for this phylogroup has been low to date.

Culture-based analysis of follicular casts from a small sample of acne (*n* = 12) and healthy (*n* = 11) subjects has also shown that multiple clones (up to 10) may coexist within the same follicle, but for most subjects one ST appears to dominate [65]. In acne patients, type IA_1_ CC1 STs were mostly present, while healthy follicles had a more heterogeneous clonal composition being dominated by clones representing phylogroups IA_1_, IA_2_, IB and II; this is broadly consistent with the data from the MLST_8_ isolate database (Figure 4). This study again highlighted that the clonal composition of surface skin may not always reflect the composition of the sebaceous follicle.

#### 3.3.2. 16S rDNA Metagenomic Analysis

A 16s rDNA-based metagenomics approach for the study of *P. acnes* strain population structures associated with acneic and healthy skin has been described by Fitz-Gibbon et al. [66]. Such an approach offers distinct advantages to traditional culture-based methods that may be unintentionally biased towards the isolation of specific strain types. This study largely focused on sampling the pre-lesional follicular microbiome on the nose of subjects (where lesions are normally not present), so as to minimise any variation due to skin site differences. Amplification and cloning of 16s rDNA from the pilosebaceous units of 49 patients with acne, and 52 normal controls, followed by Sanger sequencing of nearly full length sequences, revealed *P. acnes* to be the dominant species within sebaceous follicles, representing 87% of all clones; other species present were *Staphylococcus epidermidis*, *Propionibacterium granulosum* and *Propionibacterium humerusii*. While no statistically significant difference was found in the relative abundance of *P. acnes* between acne and control subjects, differences were identified in the strain populations when the 16S rDNA sequences were ribotyped (Table 1).

A total of six major ribotypes (RT) were predominately associated with acne patients (RT4, RT5, RT7, RT8, RT9 and RT10), with four of these ribotypes demonstrating statistically significant enrichment in acne subjects (RT4, RT5, RT8 and RT10) (Table 1). Furthermore, a subset of type II ribotypes (RT6) revealed a statistically significantly enrichment in healthy skin (Table 1). A total of 3±2 ribotypes were associated with each individual, and strains isolated from the same individuals were more closely related than those isolated from other subjects, thus suggesting clonal expansions within each individual microbiome [66].

Despite differences in sampling approaches and methodology used, and problems with detailed comparison of the results obtained from ribotyping and MLST due to a lack of equivalency in resolving power (the 16S locus is very highly conserved with only a small number of type-specific SNPs) [27,67], the data from the 16S rDNA metagenome generally matches those from culture-based MLST investigations with respect to the association of specific type IA_1_ CCs with acne (Table 1) [21,22,27]. In particular, RT4 and RT5 which are significantly enriched within acne patients predominately reflect CC3 (type IA_1_) and all CC107 (type IC) analysed to date, while RT8 is characteristic of CC4 (type IA_1_) (Table 1). MLST studies have also found a clear association of CC1 STs (type IA_1_) with acne [21,22,27] but, based on 16S rDNA metagenome data, the majority of these STs, which equate to RT1, are not statistically linked to the condition [66]. One explanation for this discrepancy may reflect the fact that RT1 is also representative of CC5 strains (type IB) which are not acne-associated but common on normal skin, potentially skewing the results for the control population. Strains from the type IA_2_ lineage, not found to be acne-associated by culture-based MLST analyses [21,22,27], were also not associated with the condition based on 16S rDNA metagenomics and were more frequent on normal skin, although this did not quite meet statistical significance (Table 1) [66].

An interesting observation that arose from this metagenome study was the association of the type II genotype RT6 with healthy skin; this ribotype corresponds to CC6. Apart from the ST26 lineage, however, STs from this CC have also been cultivated from clinical samples, including prostate tumours, prosthetic joint infections, corneal scrape and blood samples [27]. Furthermore, type II STs from CC72, which were previously described only in association with healthy skin based on MLST analysis [22], have now also been isolated from clinical specimens, although their exact relevance is unknown in these contexts. These associations would tentatively suggest a potential role for type IIs in skin health, but also a capacity to cause opportunistic infection under the correct circumstances; such potential causal effects will, however, require further studies for confirmation. It was also very interesting to note that type II RT2 strains, although not statistically associated with acne, were approximately equal in abundance on the skin of acne patients versus controls (based on the percentage of clones), but yet appear infrequently isolated from acneic skin based on earlier culture-based studies [21,22,27]. This could potentially reflect ethnic differences in the populations sampled and the grade or severity of the condition [65]. Also, IFM analysis of skin biopsy samples with monoclonal antibodies (MAbs) has shown the presence of both type IA and type II within the sebaceous follicles of both acne and control subjects, with evidence that type II is more predominant upon semi-quantitative analysis of selected samples [44].

#### 3.3.3. Shotgun Metagenomic Analysis

In a follow-up study from the same group, Barnard et al. [68] used ultra-deep metagenome shotgun sequencing to further characterise their original acne and control cohorts at the taxonomic as well as at the functional level, but also included samples taken from healthy subjects aged 55–79 years. *P. acnes* was found to be slightly more abundant within healthy skin versus acneic skin. From a population genetic perspective, RT4, RT5 and RT8 were again found to be more abundant and prevalent in acne patients, while RT6 was more prevalent in healthy individuals. Some new observations from this study were the increased levels of *P. granulosum* in healthy subjects versus acne patients, and the influence of the relative abundances of *P. acnes* and *P. granulosum* on the clinical state of the skin. A positive correlation between *P. acnes* bacteriophage abundance and healthy skin was also determined, indicating a potential role of phages in modulating the follicular populations of *P. acnes* during skin health and disease. Of most interest was data demonstrating that the population genetic composition of *P. acnes* in older individuals, who are no longer susceptible to acne, was similar to that found in younger healthy subjects; RT1, RT2 and RT3 were present, but RT4, RT5 and RT8 were absent [68].

#### 3.3.4. Antibiotic Resistance

The widespread use of oral and topical antibiotics to treat acne, and other chronic skin conditions like rosacea (where *P. acnes* is not involved), often for months at a time, has led to the worldwide emergence of *P. acnes* strains primarily resistant to tetracyclines, macrolides and lincosamines [69]. This resistance is primarily mediated via mutations within the rRNA operon; *P. acnes* is especially well placed for this mechanism, as it only has three copies of the operon with no evidence of heterogeneity. While tetracycline resistance results from a single nucleotide polymorphism (SNP) within the 16S rRNA gene (G1058C), resistance to macrolide-lincosamide-streptogramin B (MLS) antibiotics, including erythromycin and clindamycin, is associated with different SNPs in the 23S rRNA locus (e.g., G2057A; A2058G; A2059G) [70]. Other mechanisms of resistance include the presence of the *erm*(X) gene which is located on a mobile genetic element [71].

**Figure 7:**
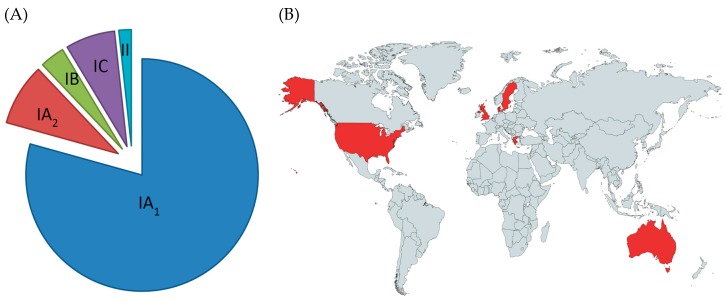
Association of antibiotic resistance with *P. acnes* phylogroups (**A**). Data is combined from the MLST_8_ studies of McDowell et al. [22] and Giannopoulos et al. [74]. Countries to date in which inter-continental spread of multi-resistant forms of the ST3 lineage (CC3) of *P. acnes* have been reported based on MLST analysis (**B**) (other countries are also likely to contain this multi-resistant lineage).

Early attempts to understand the relationship between antibiotic resistance and specific *P. acnes* strain types were based on pulse field gel electrophoresis analysis [70,72]. This provided evidence that different resistance genotypes were distributed throughout Europe among patients with acne and other infection types. Furthermore, a clonal distribution was observed for strains carrying the 16S rRNA SNP associated with tetracycline resistance, while no correlation was found between genotype and 23s RNA SNPs conferring resistance to clindamycin and erythromycin. As these studies were conducted before the MLST and WGS population genetic structure of *P. acnes* was known, interpretation of the data in relation to CCs and STs has been limited. Subsequent MLST, SLST and ribotying studies have, however, now revealed that resistant strains primarily, although not exclusively, belong to the type IA_1_ clade (CC1 and CC3) [22,64,66,73,74] (Figure 7A). In particular, the ST3 lineage (RT4) appears overrepresented in many of these studies, being associated with high level resistance to tetracycline, erythromycin and clindamycin. Furthermore, resistant forms of this genotype have been found worldwide (Figure 7B) and can also be found on the skin of healthy individuals who have not been treated with antibiotics, indicating its circulation within the human population [22]. Another interesting observation from these molecular epidemiology studies has been the association of antibiotic resistance with the type IC clade [22]. While only a small number of these strains have been described, they have so far all contained SNPs in both the 16S and 23S rRNA genes associated with tetracycline and MLS antibiotic resistance [22]. Further studies will, however, be required to determine whether antibiotic resistance is a defining characteristic of this phylogroup. While originally isolated from patients with acne, this clade has now also been recovered from normal skin [75]. Resistance has also been identified in strains from the type IA_2_, IB and II phylogroups, but at much smaller rates [22,63,64,74].

### 3.4. Insights into the Role of P. acnes in Acne from Comparative Genomics

**Figure 8:**
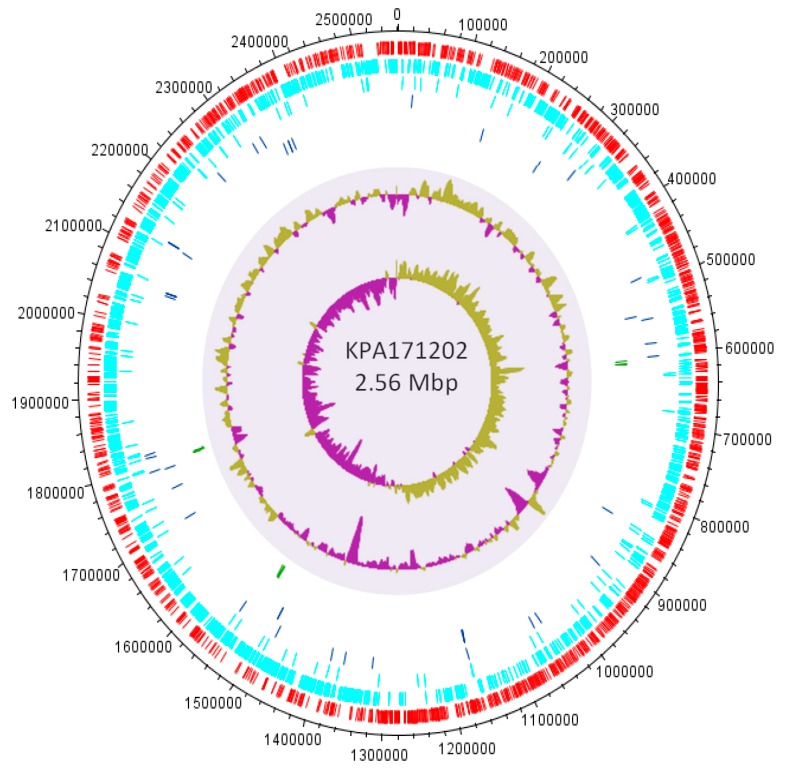
Circular map of the KPA171202 genome. From the outside to the centre: ORFs in the positive (red) and negative (green) strands, pseudogenes, tRNA (blue), rRNA (green), %GC plot and GC skew (purple and green equates to negative and positive values, respectively).

The first *P. acnes* genome sequence, representing the type IB strain KPA171202 (agar plate contaminant; ST5; CC5), was described in 2004 [76] (Figure 8). Since then, over 190 complete and draft genome sequences are now available for the bacterium. The average genome size is 2.50 Mb, with a GC content of 60% and approximately 2500 open reading frames (ORFs) [66,67,76].

Initial analysis of the KPA171202 genome sequence revealed the pathogenic potential of the organism, with many features that help to explain its lifestyle adaptions and capacity to induce inflammation and tissue damage [76]. In particular, genes for putative tissue degrading factors, including haemolysins, co-haemolytic Christie-Atkins-Munch-Peterson (CAMP) factors, sialidases, endoglycoceramidases, lipases, porphyrins and hyaluronate lyase (HYL) are present, as well as genes for cell wall-/cell envelope-associated proteins with inflammatory potential, and proteins that contain multiple proline-threonine repeats (PTRs) (Table 2). Also present are genes containing nucleotide repeat sequences or homopolymeric tracts (HPTs) of guanine or cytosine residues within 5’ untranslated regions, or within coding sequences [76,77]. These contiguous stretches, which are generated via slipped strand mispairing, are signatures of antigenic or phase variation thus modulating interaction of the bacterium with the host.

WGS analysis has provided important insights into the pathogenic potential of *P. acnes*, and provided a platform from which new evidence-based studies can be designed and conducted. The historic view that *P. acnes* is benign and relatively unimportant as a human pathogen is now discredited, but much work still lies ahead in understanding its exact role in a range of human diseases. With the growing number of multiple whole genome sequences of *P. acnes* now available, one key area for investigation has been to determine how the different phylogroups vary at the genomic level, especially in respect to putative virulence factors, and whether this could help to explain potential functional differences and the association of type IA_1_ strains with acne.

#### 3.4.1. Genomic Islands and a Flexible Gene Pool

**Figure 9:**
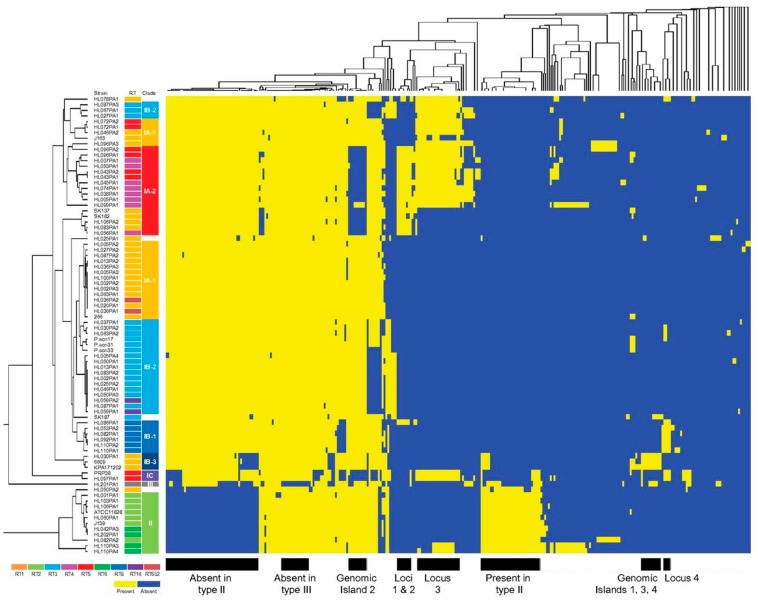
Clustering of noncore genomic regions present in 82 *P. acnes* genomes. Rows represent genomes, and columns represent 314 noncore regions that are longer than 500 bp. The presence of a noncore region is coloured in yellow, and the absence is coloured in blue. Taken from Tomida et al. [67].

The *P. acnes* chromosome appears highly stable, with a conserved synteny, and a closed pan-genome. Analysis of 82 genome sequences has shown the total length of noncore regions to be approximately 0.90 Mb [67]. While the overall plasticity of the genome is low, within the accessory genome several regions have been identified which display evidence of horizontal gene transfer (HGT), including aberrant G+C content and flanking insertion sequences, and are differentially found between phylogroups [67,78,79,80]. Most importantly, the island-like regions which contribute to this flexible gene pool encode a range of putative virulence determinants and fitness factors [80]. Of particular interest are a small number of genetic elements, designated genomic loci 1, 2, 3 and 4, that are present in specific acne-associated lineages and, therefore, may prove to be important in acne pathogenesis [66,68,79,80] (Figure 9).

Briefly, genomic locus 1 contains prophage-related genes, while genomic locus 2 (CC3; RT4 and RT5) spans over 20 kb and encodes 23 ORFs, including a cluster of Streptolysin S-associated genes (*sag*) which are involved in the biosynthesis and transport of a bacteriocin. Other genes present in this locus include those with putative roles in virulence, cell viability and self-immunity. Locus 3 (CC1, CC2, CC3, CC107) is present on a 55 kb linear plasmid that encodes a tight adhesion (TAD) locus with homology to the tight adherence genes of *Aggregatibacter actinomycetemcomitans*, and which may be important for host colonization. Subsequent metagenomic studies have confirmed a higher relative abundance and prevalence of all three loci in acne patients versus healthy controls; indeed, locus 2 appears rarely found in the microbiome of healthy skin [68]. A fourth unique locus (locus 4) of 20kb in length is present in strains from CC4 (RT8) (Figure 9). This locus encodes a series of nonribosomal peptide synthetases that may enhance pathogenicity, as well as genes involved in the synthesis of a cyclic lipopeptide. Genomic analysis of a small number of CC1 isolates from acneic and healthy follicles has also shown no differences in group-specific genes, plasmid content or sequences that may affect gene expression (promoters and homopolymeric tracts) [65]; this therefore highlights the importance of the host response as a contributing factor in the development of acne.

In relation to other phylogroups, type IA_2_ strains (RT3 and RT16) appear to have fewer non-core regions which may reflect a lack of rearrangement hot spot family proteins which function in genomic rearrangements [67]. The type IB phylogroup, which is a tight phylogenetic cluster, also contains a genomic region that appears to be a cryptic prophage [78]. This island has been inserted into a gene for a type II restriction enzyme, with the resulting gene fragments flanking the region. Type IB strains, and the aberrant type IA strain SK187, also contain an island with a region encoding a thiopeptide antibiotic, which targets Gram-positive bacteria [78]. This island is also present in type III strains, and may be a hotspot for insertion/deletions [77]. The large type II phylogroup also displays several genomic differences when compared to type I strains, including the deletion of genes involved in iron uptake which may impact on survival under low iron conditions; this region is also missing from type IC strains [77]. Inspection of genome sequences for type II strains has also identified insertions and deletions in putative lipase genes compared to type I strains, which may help to explain previous reports of reduced lipase activity within this group and their lack of association with acne [67].

As type III strains have never been recovered from acneic skin, comparative analysis of their noncore regions to acne-associated strains could prove valuable to filter and identify genes and genetic elements that may be relevant to acne virulence and skin health. Analysis of four publicly available type III genomes has shown the presence of eight noncore regions specific to this phylogroup, and a further two regions which are present in three of the four genomes [10]. These regions contain ORFs with putative functions in transcriptional regulation and transport, metabolism, phage proteins and hypotheticals. One interesting observation was the presence of a region containing putative type III secretion system (T2SS) genes in close proximity to a putative tight adherence (Tad) locus, although unrelated to the plasmid Tad locus described in type IA_1_ strains associated with acne [66]. Regions present in type I and II strains, but missing from type III, contain ORFs for dimethyl sulfoxide reductase (which may influence anaerobic growth), bacteriocins, iron and other ABC transporter systems, HYL and cobalamin biosynthesis [10]. For more detailed analysis of indels within *P. acnes* the reader is referred to Scholz et al. [77].

#### 3.4.2. CRISPR/Cas

One of the most striking and important differences between phylogroups at the genomic level relates to the clustered regularly interspaced short palindromic repeats (CRISPR)/Cas locus. A complete set of the CRISPR/Cas genes, which provides “adaptive immunity” against phages and plasmids, is located on a 16 Kb genomic-like island exclusively present in almost all type II strains [66,81]. Within type I and III phylogroups, however, only remnants or fragments of the system exist due to partial deletion events [80,81]. Indeed, the presence of this active CRISPR/Cas system was the basis for the name “*defendens*” (defending, guarding, protecting) in the recent proposal of this phylogroup as a distinct subspecies [24]. These observations regarding the CRISPR/Cas locus are important as their absence provides a mechanism for more rapid evolution of the type I and III clades via HGT of foreign mobile elements containing virulence and fitness traits [66,81]. Interestingly, sequence analysis of CRISPR spacers among multiple type II strains has revealed previous challenges with genomic loci 2 and 3, present in a subset of type I strains, as well as *P. acnes*-specific phages [66,81]. The type II phylogroup behaves as a single clade in terms of shared indels, and this may also reflect the presence of an active CRISPR/Cas system [77].

Analysis of CRISPR/cas shared spacer sequences also provides an opportunity to gain further insight into the evolution of this bacterium. The results from this analysis are consistent with phylogenetic relationships based on whole genome SNP clustering [67]. Also, the presence of CRISPR/Cas remnants within the type I and III phylogroups provides circumstantial evidence that they may be younger sub-populations derived from the type II division which represents a more ancient lineage [80,81].

#### 3.4.3. Homopolymeric Tracts (HPTs)

The presence of HPTs in *P. acnes* gives the bacterium flexibility in its capacity to control gene expression, thus responding to local ecological changes. Using 90 genomes spanning the known diversity of *P. acnes*, Scholz et al. [77] examined the HPTs within *P. acnes* and their distribution within different phylogenetic clades. A total of 54 polymorphic HPTs were identified (nearly all being G or C repeats) between genes or within ORFs. A correlation between repeat variation and different clades was observed, indicating HPT changes are uncommon and relatively stable, thus accumulating over a long time frame.

**Figure 10:**
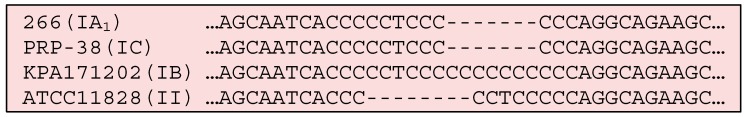
Homopolymeric C tract within the 5’ end of the ORF for DsA1 (PPA2127) leading to putative differences in phylogroup expression due to frameshift mutations.

HPT variation effected gene expression, highlighting how these sequences could influence the functional activity of the different phylogroups, and the expression of genes associated with putative virulence factors. These may prove important in the context of acne. An excellent example of this is found with the gene sequence for the putative adhesin and fibrinogen-binding protein DsA1 (PPA2127), which has a long homopolymeric C tract at its 5’ end, PTRs within its sequence, and is a putative MSCRAMM (microbial surface components recognising adhesive matrix molecule) (Figure 10) [31,32]. This gene encodes a functional protein in type IA and IC strains only, but not type IB, II or III [19,20,32,82]; also, in KPA171202 the start codon is mutated (Figure 10).

### 3.5. Insights into the Role of P. acnes in Acne from Transcriptomic and Proteomic Analysis of Phylogroups

#### 3.5.1. Transcriptome

While inspection of the genome provides some insight into potential functional differences between phylogroups in the context of health and disease, it provides no information regarding variation at the transcriptional and translational levels. To address this issue, several studies have also compared differences between the major genetic divisions of *P. acnes* based on their transcriptomic and proteomic properties.

Differences at the transcriptome level between strain types representing acne- and non-acne associated phylogroups has been described by Brzuskiewicz et al. [78] using a whole genome microarray derived from strain KPA171202 (type IB). After growth in brain heart infusion broth (BHI), comparison of the KPA171202 transcriptome with the type IA_1_ strain 266 (pleuropulmonary infection; ST20, CC1) revealed differential expression of 119 common genes during mid-log phase. These included genes for several putative virulence factors, including triacylglycerol lipase (PPA2105), endoglycoceramidase (PPA0644) and DsA1 (PPA2127), which were all upregulated in 266 (2.5-, 4- and 6-fold, respectively). A putative haemolysin (PPA0565) and the immunoreactive iron acquisition protein *htaA* (PPA0786) were also found to be upregulated in 266; in the latter case this may be important for survival and growth in iron limited conditions. In contrast, CAMP factor 4 and lysozyme activity were upregulated in KPA171202 (8-and 12-fold, respectively); CAMP factor 4 was one of the most strongly expressed proteins irrespective of growth phase. Other expression differences which were observed between these distinct lineages related to energy metabolism and protein biosynthesis with strain 266 possessing an extended anaerobic metabolic versatility for fermentative pathways that utilise amino acids.

Differential and global RNA-seq analysis has also revealed pervasive transcription within KPA171202, with an extensive array of riboswitches, leaderless mRNAs, small non-protein-coding RNAs and vegetative promoters in gene regulation, although how these differ between phylogroups in relation to expression profiles associated with disease is currently not clear [83].

#### 3.5.2. Proteome

Whole cell proteomic profiling of *P. acnes* via SELDI-TOF mass spectrometry (after anaerobic growth on blood agar) has identified four distinct intraspecies proteomic groups (A–D) at a 60% similarity level that correspond broadly to the major phylogroups [84]; whole cell protein analysis is an important component of a polyphasic taxonomic approach and correlates well with DNA-DNA hybridization results [85]. Group A contains type IA and IB strains, group B is exclusively type III strains, group C is a combination of type IA_2_, IB and II strains, while group D is mostly type II strains with a small number of type IA_2_ (type IC was not analysed). Using a type IB strain that could grow under different oxygen tensions, or in its absence, this study also showed that protein expression under anaerobic or microaerophilic conditions was similar, but distinct from that produced during aerobic growth. More generally, this study highlighted clear differences between the major genetic divisions in terms of their proteome.

Proteomic comparison of the secretome (mid-exponential phase) from *P. acnes* strains representing different phylogroups (types IA_1_, IB, II and III) was carried out by Holland et al. [82] after growth in BHI; many of these secreted proteins also appear to comprise extracellular vesicles [86]. A set of 20 proteins was found to be produced by the majority of the strains tested, and eight proteins by all the strains. These included proteins with putative hydrolytic properties, such as endo-glycoceramidases (PPA0644; PPA2106), lysozyme (PPA1612), a protease (PPA1310), peptidase/glycosyl hydrolyase (PPA2239), the triacylglycerol lipase GehA (PPA2105), which is believed to hydrolyse sebum triacylglycerides, and hypotheticals (PPA0533; PPA0533; PPA1939). Key phylogroup differences included reduced lysozyme and increased GehA secretion by type IA_1_ (266) versus type IB (KPA171202), and exclusive production of an outer membrane protein (OmpA; PPA0263) and DsA1 (PPA2127) by strain 266; this was consistent with transcriptomic data [78]. Interestingly, the protein DsA2 (PPA2210), which is very similar to DsA1 with respect to the presence of HPTs and PTRs, was only produced in stationary phase. These differences, especially in relation to differential production of GehA, DsA1 and DsA2 proteins, may be particularly relevant in the context of acne pathogenesis.

The secretion of DsA1 and DsA2 by type IA strains only is also in agreement with earlier studies based on monoclonal antibody (MAb) typing [19,20]. Using a MAb (QUBPa1) which was subsequently revealed to target DsA1 and DsA2 antigens on the cell surface, only strains representing type IA_1_, IA_2_ and also IC were immunoreactive displaying both polar and septal labelling. Furthermore, some cells derived from a clonal strain did not show any reactivity tentatively indicating within strain phase variation in expression. We can speculate that the potential for type IAs to modulate their interaction with the host immune system via the DsA immunogenic proteins may be important in the recurring nature of acne.

*P. acnes* is relatively unusual in that it contains a family of five CAMP factor homologue genes which encode putative co-haemolysins; although these proteins may also bind immunoglobulins and function as pore-forming toxins [30,76]. Interestingly, all phylogroups tested in the study by Holland et al. [82] were found to produce the CAMP factor 2 (PPA0687) protein which is under consideration as a vaccine target (see Section 3.9.3), but only KPA171202 was found to produce CAMP factor 4 (PPA1231) in line with previous transcriptomic and proteomic data which demonstrated its upregulation in this strain [78,82]. Of particular note was the observation that CAMP factor 1 protein production was not detected, even though previous Western blotting experiments with anti-CAMP factor sera revealed that this protein is produced in abundance by type IB and type II strains (after anaerobic growth on blood agar) [30]; interestingly, significant expression levels of the CAMP factor 1 gene have also been observed in KPA170202 during exponential phase growth [78]. Intriguingly, although produced in large amounts by types IB and II, which are not considered to be important in acne pathogenesis, the CAMP factor 1 protein was observed in sebaceous follicles of both acne and healthy controls, although its significance here is not yet known [44]. Furthermore, a recent study has also demonstrated that CAMP factor 1 recognises TLR-2 and stimulates IL-8 production which indicates it may play a role in the inflammatory response to the bacterium [87] (see Section 3.7). Western blot analysis has also revealed that CAMP factor 4 appears more highly expressed by type IB and II strains, while type IA produces higher quantities of CAMP factor 2 [30]. Differences between studies in regard to CAMP factor production may reflect variation in the growth conditions used (broth vs solid media). While it is not clear what exact role, if any, the CAMP factor family play in acne, the differential production of the proteins between phylogroups warrants further studies to better understand their importance.

**Figure 11:**
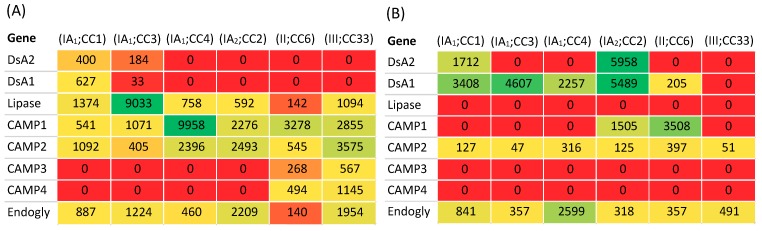
Key secreted (**A**) and cell-wall (**B**) proteins (fmol/µg total protein) produced by representative strains of phylogroups IA_1_ (CC1, CC3, CC4), IA_2_, II and III. Organisms were grown in reinforced clostridial media before mass spectrometry analysis. Secreted and cell wall protein concentration data is taken from the study of Yu et al. [51]. Endogly = Endoglycoceramidase. HL005PA1 (normal skin; ST11, CC1), HL043PA1 (acne; ST3, CC3), HL110PA1 (acne; ST4, CC4), HL013PA1 (acne; ST2, CC2), HL110PA4 (acne; ST7, CC6), Asn12 (cervical disc; ST33, CC33).

Analysis of the *P. acnes* secretome also identified the production of a secreted HYL (PPA0380) by type IB (KPA171202) and II strains (strain 329), but not other phylogroups [82]. In a follow-up transcriptomic and biochemical investigation of this observation, Nazipi et al. [88] revealed that *P. acnes* employs two variants of HLY, one of which is highly active in type IB and II strains resulting in complete degradation of hyaluronic acid (HA), and another present in type IA strains which has lower activity resulting in incomplete degradation of HA and the generation of low molecular weight (LMW) fragments. The HLY gene in types I and II was also superior at degrading other glycosoaminoglycans. The HLY gene is not present in strains from the type III phylogroup [77,88]. In the context of disease, the HLY of type IB and II strains may be important for degradation of glycoaminoglycans within dermal and epidermal extracellular matrix, facilitating tissue spread and bacterial dissemination. Type IB and II strains appear more frequently associated with soft and deep tissue infections, although their clinical relevance in such circumstances is not always clear [27]. In contrast, in acne the incomplete degradation of HA by type IA_1_ strains may result in the production of pro-inflammatory LMW fragments after comedone rupture and exposure of the underlying dermis [88]. These fragments could activate macrophages and dendritic cells via Toll-like receptor 2 and 4 signalling [89,90]. Other proteins which appeared confined to the type IB and type II secretome were purine nucleoside phosphorylase (PPA2097), a putative lysophosphlipase (PPA2142) and a hypothetical protein with SH3 and RlpA domains (PPA2175) [82]. Further proteomic studies by Yu et al. [51] also investigated the differential production of secreted and cell wall proteins among representative type IA_1_, IA_2_ and II strains from acneic and normal skin (HL005PA1, HL043PA1, HL110PA1, HL013PA1, HL110PA4), and also included the type III strain Asn12 (Figure 11).

Consistent with previous studies, DsA1 and DsA2 were found to be produced by type IA strains only, although variability in secreted and cell wall production between type IA_1_ CCs was observed (Figure 11). CAMP factor 2 was secreted by all phylogroup strains examined, especially type III, although some also appeared cell wall-associated (Figure 11). CAMP factor 1 was produced in much greater amounts (both secreted and cell wall-associated) by type II compared to CC1, consistent with that described by Valanne et al. based on Western blotting [30] but, interestingly, secreted levels were the highest with the representative CC4 strain, while the type IA_2_ and type III strains also produced appreciable amounts of the protein; strains from these phylogroups were not examined by Valanne et al. [30] (Figure 11). CAMP factors 3 and 4 were only produced by type II and III strains, albeit at relatively low levels [51].

### 3.6. Insights into the Role of P. acnes in Acne from Other Biochemical Studies of Phylogroups

It has been known for some time that type I strains show variable β-haemolysis activity, which is absent from type II and III strains [34,91]. Although not described in previous proteomic and transcriptomic studies, biochemical analysis has also found that *P. acnes* produces neuraminidase or sialidase activities that cleave sialoglycoconjugates [92]; several putative genes for the enzyme have been identified within the genome sequence (PPA1560, PPA684, PPA685). On the basis of data from Lomholt and Kilian [21], this activity appears confined to organisms from the type IA phylogroup only (type IC not described), but data from Niazi et al. [13] has also suggested neuraminidase activity can be found in a small proportion of type IB, II and III strains. These enzymes could function as putative virulence factors, and may also use the cleaved sialic acid as a carbon source.

**Figure 12:**
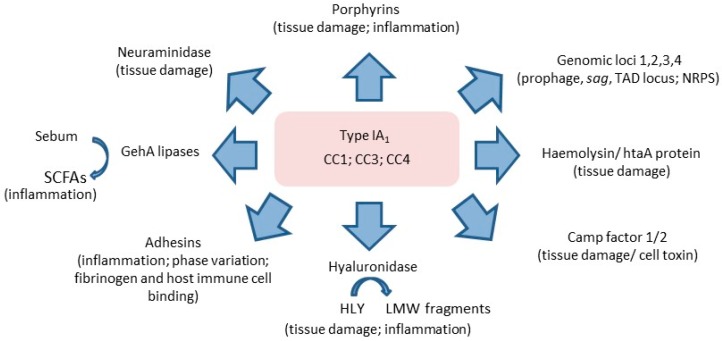
Summary of key virulence determinants expressed by type IA_1_ strains that, collectively, may help to explain, alongside host response, their dominant association with acne versus other phylogroups. Data is compiled from genomic, proteomic, transcriptomic and biochemical studies.

*P. acnes* is also known to produce pro-inflammatory porphyrins which may be important in skin tissue damage via the generation of free radicals [76,93]. The production of porphyrins is also enhanced with vitamin B_12_ supplementation [see Section 3.8]. Porphyrin levels are higher in acneic versus healthy skin, but are reduced in patients who positively respond to treatment [94]. At the intraspecies level, differences between acne- and non-acne-associated ribotypes in the production of porphyrins have recently been described [95]. Acne-associated type IA_1_ RT4 and RT5 strains (CC3) were found to produce significantly higher levels of porphyrins compared to health-associated type II RT2 and RT6 strains, and this was further enhanced with vitamin B_12_ supplementation. Furthermore, a repressor gene of porphyrin biosynthesis (*deoR*) was identified, and was found to be present in all RT2 and RT6 strains but not in acne-associated RT4 and RT5. Interestingly, although porphyrin production was not investigated in relation to other type IA_1_ strains, or strains from the type IA_2_, IB, IC or III phylogroups, the *deoR* repressor gene was also shown to be missing in type IA_1_ CC1 and CC4 strains, but present in the small number of type IB, IC and III strains examined. While the latter is consistent with types IB and III not being associated with acne, it suggests that acne-associated type IC strains may also not produce these metabolites. A summary of the key virulence determinants that collectively may help to explain the association of type IA_1_ strains with acne is highlighted in Figure 12.

### 3.7. Insights into the Role of P. acnes in Acne from Cell-Based and Skin-Explant Studies

A key aspect of acne research has been understanding how *P. acnes* interacts with host cells that are present in the pilosebaceous follicle (keratinocytes, sebocytes and macrophages), and stimulates an immune/inflammatory response that contributes to the pathogenesis of the disease [28,29,51,60,96]. While it is well known that *P. acnes* can stimulate a wide range of inflammatory responses from such cells, it is only now, with our increased understanding of the bacterium’s intraspecies diversity, that we can start to tease apart how this differs between phylogroups and specific strains that are associated with acne and skin health.

One of the first studies to investigate the effect of different phylogroups (types IA_1_, IB, II) on the innate immune response of human epidermal keratinocytes was carried out by Nagy et al. [28]; this work was originally conducted before MLST analyses identified specific type IA_1_ CCs and STs associated with acne. This study illustrated the differential response of host cells to different strain types, with the type IA_1_ strains 889 (endocarditis; ST1, CC1) and 2005 inducing human β-defensin-2 (hBD2) and involucrin gene expression, while non-acneic type IB strain 6609 (normal skin; ST5, CC5) and type II strain ATCC11828 (subcutaneous abscess; ST27, singleton) had no effect; the hBD2 protein is an important host defence molecule and chemotactic agent for immature dendritic cells, memory T cells and neutrophils. The type IA_1_ strain 889 also appeared capable of enhancing keratinocyte growth in vitro. Furthermore, all four strains induced IL-8 gene expression and the production of both hBD2 and IL-8 appeared TLR2 and TLR4-dependent [28].

In a follow-up study, the effect of these strains on immortalised sebocytes (SZ95) was also investigated [29]. Type IA_1_ strain 889 and type IB strain 6609 significantly induced hBD2 expression, but the type II strain ATCC11828 had no effect; these results were confirmed by immunostaining. Both type IA_1_ and type IB strains also enhanced IL-8 and TNFα gene expression, but IL-1α, TLR2 and TLR4 gene expression was not affected; they also differentially modulated the viability of SZ95 sebocytes, but this was much higher with the type IB strain. While these studies clearly illustrate the capacity of different *P. acnes* strain types to stimulate an innate/inflammatory response from host cells present in the pilosebaceous follicle, they are limited by the small number of host genes and strains examined.

Further investigation of the effects of different phylogroup strains, representing types IA_1_, IB, IC, II and III, on cutaneous innate immunity was carried by Jasson et al. [96] using ex vivo skin explants (from abdominal skin) and immunochemistry, RT-qPCR and ELISA analysis. These explants were treated with membrane-fraction lysate and the results compared to an LPS-treated control. One interesting observation was that the type III isolate Asn12 appeared the most pro-inflammatory of the strains, in particular stimulating TNFα, MMP-13 and TIMP-2, as well as protease activator receptor 2 (PAR-2). In contrast, the normal skin-derived type IB strain 6609 was classified as the least pro-inflammatory of the strains examined, although consistent evidence of TLR2, MMP-13 and TIMP2 up-regulation was observed with different detection methods. The other strains examined, which included the acneic type IA_1_ isolate PRP-60 (ST20, CC1), the acneic type IC isolate PRP-38 (ST70, CC107) and the type II isolate ATCC11828, also stimulated an inflammatory response although this was variable and the results appeared to depend on the method of analysis. On the basis of the results, the pro-inflammatory potential of the strains examined was ranked as type III (strongest), type II, type IC, type IA_1_ and type IB (weakest).

The inflammatory potential of different *P. acnes* phylogroups (including strains containing a large acne-associated plasmid, p) on PBMCs was examined by Yu et al. [51] (also see 3.5.2). They found that certain type IA_1_ (CC3 p+, CC4) and type IC (CC107) acne-associated lineages, as well a subset of non-acne-associated or neutral type IB (CC5) and II lineages (CC6, CC72) (subset 1), induced 2- to 10-fold higher levels of inflammatory IFN-y compared to other type II (CC6) and type III strains (CC77) found to be positively associated with healthy skin, as well as a further non-acne-associated type IA_1_ (CC1; CC3 p-) and type IA_2_ (CC2) subset (subset 2). In contrast, this latter subset 2 group of non-acne-associated strains induced nearly 2-fold higher levels of IL-17 than acne-associated lineages, which themselves induced 2-fold higher levels of IL-17 compared to other phylogroups. Furthermore, skin health-associated type II and III lineages and neutral subset 1 strains induced 2-to-4 times higher levels of anti-inflammatory IL-10 compared to acne-associated strains and neutral subset 2 strains. Collectively, this has led to the suggestion that acne-associated phylogroups may have increased potential to induce acne via induction of Th1 and Th17 responses, and that lineages currently classified as non-acne-associated or neutral with regards to their presence on both acne and healthy subjects may also be associated with mainly Th1 responses, despite counterbalancing IL-10 induction, or Th17 responses. There is recent literature that indicates specific individual acne cases may be driven by strains outside the strongly acne-associated type IA_1_ grouping, such as type IB and IA_2_ [63,64]. In all these studies, while the variability of the host immune response between individuals was high, the relative patterns of response were found to be largely similar.

In a recent follow-up study from the same group, Agak et al. [58] developed this work by demonstrating that acne-associated type IA_1_ (CC3 and CC4) and skin health-associated type II (CC6) *P. acnes* lineages also differentially modulate CD4+ T-cell responses, leading to Th17 cells of varied phenotype and function. Most interestingly, clones derived from skin health-associated strains produced antimicrobial activity against *P. acnes* with complete breaches in the cell wall envelope, while those from acne-associated strains were lacking in this ability. This suggests that Th17 cells can be either pathogenic or protective in the context of acne pathogenesis depending upon the nature of the *P. acnes* lineage causing induction. While it remains to be determined whether certain strains of *P. acnes* do indeed induce an immunologically protective effect that promotes skin health, such work does highlight the complex nature of the host-microbiota interactions within the skin.

Analysis of human keratinocytes (cell line HaCaT) has also found that CAMP factor 1 induces IL-8 production via interaction with TLR2 [87]. This interaction was found to be the strongest with a range of type IB and II strains, consistent with previous reports that these phylogroups produce large quantities of the protein [30]. In contrast, strains of type IA_1_ and IA_2_ produced little or no CAMP1-TLR2 binding or IL-8. While the exact role this interaction plays in acne pathogenesis remains unclear, specifically because type IB and II strains are generally not acne-associated, it may be relevant to other types of opportunistic infections where these lineages are potentially more important.

One caveat with cell-based studies, as well as different omic comparisons of *P. acnes*, is the relatively small number of isolates analysed from each phylogroup, combined with the different strains examined and methodologies used. This makes it difficult to directly compare studies and draw definitive conclusions regarding host immunological, and other, responses to the different *P. acnes* phylogroups; it may also explain some of the contradictory results obtained. While intra-phylogroup variation may be less of a consideration for strains of types IA_2_, IB, IC and III that appear relatively conserved at the phylogenetic and accessory genome level, for type IA_1_ and type II phylogroups, which display deeper phylogenetic structures, a wider range of STs should be examined. Furthermore, in vitro models of infection provide, at best, limited insight into the true nature of the host response which is a complex and intricate interaction involving multiple cell types, biochemical pathways and immunological cascades.

### 3.8. Insights into the Role of P. acnes in Acne from Meta-Transcriptomic and Meta-Proteomic Analysis of Follicular Microbiota

To understand whether the transcriptional activities of the skin microbiota contribute to acne development, Kang et al. [97] compared the gene expression profile of *P. acnes* present in the follicular contents of the nose (sampled by pore strips) of several acne (*n* = 4) and healthy subjects (*n* = 5) by RNA sequencing; *P. acnes* was the most transcriptionally abundant bacterium in the metatranscriptome compared to other organisms, including *Staphylococcus*, *Pseudomonas* and *Shigella*. A core set of 3725 operational gene units expressed in all samples, and representing different metabolic pathways encoded in *P. acnes*, were analysed. The results showed that the transcriptional activities of *P. acnes* in the skin microbiota of acne patients were distinct from those of the control subjects which formed a separate cluster. Most interestingly, the vitamin B_12_ biosynthesis pathway in *P. acnes* was found to be significantly downregulated in acne patients, suggesting that host vitamin B_12_ modulates skin microbiota activity and can contribute to acne development. When supplemented with vitamin B_12_, the expression of vitamin B_12_ biosynthesis genes also became downregulated in healthy subjects, with one individual developing acne within a week. The mechanism driving the association of vitamin B_12_ and acne is proposed to revolve around the over-production of pro-inflammatory porphyrins within the follicle as a result of repressed vitamin B_12_ biosynthesis and heightened levels of the shared substrates 2-oxoglutarate and l-glutamate, with the latter shunted away from B_12_ biosynthesis towards porphyrin biosynthesis. In contrast, when vitamin B_12_ levels are normal, the vitamin B_12_ pathway is expressed and porphyrins are produced at low levels. The observation, however, that not all individuals develop acne after vitamin B_12_ supplementation provides evidence that other host and bacterial factors regulated by vitamin B_12_ may also contribute to the development of the disease.

Meta-proteomic analysis of the contents within follicular casts (sampled by cyanoacrylate biopsies of follicular infundibula) from acne (*n* = 20) and healthy subjects (*n* = 18) were investigated by Bek-Thompson et al. [98]. This study revealed the presence of both human and bacterial proteins; the latter were exclusively from *P. acnes*. Normal casts were found to be enriched for proteins involved in protection from various stresses, including reactive oxygen species, while casts from patients with acne were enriched for proteins associated with pathways involved in host responses to bacteria, as well as tissue repair and regeneration. Some of the most notable proteins identified included myeloperoxidase, lactotransferrin, neutrophil elastase and vimentin.

Another interesting observation from this study was the much higher abundance of *P. acnes* proteins in the follicular casts from healthy volunteers versus acne patients. While it is unclear exactly why this was the case, it could reflect a reduction in *P. acnes* numbers due to the inflammatory response, or the effects of antibiotics which most of the patients were treated with [98]. Key *P. acnes* proteins abundantly found in the follicular cast proteome of both healthy and acne skin were the adhesins DsA1 and DsA2 (see Section 3.5), produced by type IA strains, as well as CAMP factors 1 and 2. In the case of CAMP factor 1, it was detected in all healthy subject’s skin, but only 10% of the acne-affected individuals. CAMP factor 2 was present in 14% of the healthy subjects, but strikingly was not detected in samples from acne patients. Endoglycoceramidases and lipolytic enzymes were also identified in health and acne-associated casts but, surprisingly, the previously described lipase GehA (PPA2105) believed to be a key bacterial virulence determinant in acne was not detected in any of the patients, and only 16% of healthy subjects. In contrast, a second and less well described lipase designated GehB (PPA1796) was detected in 50% of all healthy individuals and only 10% of acne-affected, suggesting it may play a role in skin health. Collectively, observations such as these challenge our understanding of the role *P. acnes* “virulence” determinants play in acne, and illustrate gaps in our knowledge regarding their actual role in skin health versus disease.

In addition to the fact that patients had previously been treated with antibiotics and other antimicrobials that may have impacted on their microbiota composition, other limitations of this study which may have affected the results were the nature of the sampling sites which varied between the control and acne cohorts; controls were sampled from the nose while acne patients were sampled from the face and back skin areas. Also, extracted follicular casts are heterogeneous in respect to size, composition and their state of health and disease.

### 3.9. New Therapeutic Strategies

The observation that specific acneic- and skin health-associated lineages of *P. acnes* appear to exist, alongside multi-omic, biochemical and host-microbe studies that are helping to explain the clinical relevance of such associations, will serve to create a platform for the investigation of new therapeutics to treat acne. Furthermore, such knowledge will enable us to examine and, where necessary, challenge new treatment strategies currently in development and their possible effects on commensal or beneficial lineages of *P. acnes*.

#### 3.9.1. Bacteriophage Therapy

In patients with acne, rates of *P. acnes* phage isolation from the skin are lower than from normal subjects, suggesting an important role for these viruses in maintaining the balance of skin health [67,99]. Such observations therefore highlight the potential of phage-based antimicrobial treatment to alter or shift the *P. acnes* strain composition to a more skin health-associated profile. For further detail on the biology and genetic diversity of *P. acnes* phages, and their proposal as a treatment for acne, we would refer the reader to a range of key reviews and primary research articles [100,101,102,103,104]. As indicated, one consideration when developing a treatment that targets skin microbiota is the specificity of action and effect on health-associated lineages. It has been known for a long time that type I strains of *P. acnes* appear more susceptible to phage infections compared to those from the type II phylogroup [105]. Recent studies based on our improved understanding of the population genetic structure of *P. acnes* have now enabled a more detailed analysis of this susceptibility, with strains from the type IA_1_ and IA_2_ phylogroups displaying sensitivity to infection, while those from the type IB, II and III phylogroups appear more resistant [99]. Of particular interest from the study of Liu et al. [99] was the observation that type IA_1_ strains of CC1 undergo a pseudolysogenic response, while strains from type IA_1_ CC4 and type IA_2_ strains display lysis. In contrast, Brown et al. [106] found that facial type IA_1_ strains from CC1 did undergo lysis when treated with phage that was formulated into a cetomacrogol cream and applied to lawn cultures. While phage resistance in some type II strains may reflect the presence of an active CRISPR/*cas* gene cluster (see Section 3.4.2), this does not appear to be the case for all strains and other regulatory mechanisms must also be involved in this phylogroup; this is also the case for resistant type IB and III strains which lack an active CRISPR/*cas* system [66,80,81].

As previous studies have found that type IB and III strains are not associated with acne, and strains from the type II phylogroup appear positively associated with skin health (see Section 3.3.1 and Section 3.3.2), a well-formulated lytic phage therapy may prove a viable way to selectively target acneic strains of *P. acnes* while maintaining those that are potentially beneficial components of the skin microbiota. To be effective, however, a personalised medicine-based approach may need to be adopted where an individual’s skin microbiome and *P. acnes* community structure is considered before treatment is initiated. Other issues of concern are transduction of pathogenicity genes, the presence of phage genes of currently unknown function, and the possibility that phage-resistant *P. acnes* bacteria may arise and disseminate, especially given the limited genetic diversity of *P. acnes* bacteriophages.

#### 3.9.2. Skin Probiotics

The idea or concept of using topical probiotics (masks, cleansers or creams) consisting of various bacterial formulations that will restore skin microbiome balance and selectively suppress pathogenic organisms and inflammation is a growing area of research for the treatment of acne and atopic dermatitis [107,108]. In particular, interspecies interactions and antagonism between *P. acnes* and *S. epidermidis* have been demonstrated via the production of antimicrobials and fermentation products and, in the latter case, it has been suggested that sucrose could be harnessed as a potentially novel treatment for the suppression of *P. acnes* growth via selectively augmenting fermentation by *S. epidermidis* [2,3,109,110]. While studies to date have centred on species-level competition as the basis of probiotic therapies for acne, there is currently little information on the use of commensal or health-associated lineages of *P. acnes* for the purpose of reversing or modulating “acne dysbioisis” caused by specific type IA_1_ strains; also, our understanding of the importance of *P. acnes* intraspecies competition in shaping the community structure of this bacterium within the skin is unknown. These should be areas of research which are actively pursued in the future given our newly improved understanding of *P. acnes* phylogeny. Furthermore, probiotic approaches to the treatment of acne may not prove straightforward since stable colonisation of donor bacteria is likely to be influenced by host genetics and other biological factors so that personalised probiotic therapies are likely to be required.

#### 3.9.3. Vaccine Development

The development of an acne vaccine based on targeting of *P. acnes* has long been sought since it would help to circumvent the use of antibiotic and retinoid therapies. Vaccine development has, however, been hindered by the absence of a suitable acne animal model, as well as the potential detrimental effect such a treatment may have on health-associated or beneficial strains. Under such circumstances, targeting the mechanism of virulence transition while maintaining colonisation and, therefore, commensal benefit is the ideal approach.

Recently, there has been significant interest in the development of an acne vaccine based on targeting of the CAMP factor 2 protein of *P. acnes* [111,112,113]; targeting of *P. acnes* surface sialidase has also been described but this has not been pursued [114]. Studies with a mouse model of bacterial induced inflammation have shown that CAMP factor 2 vaccination reduces *P. acnes*-induced ear swelling and production of macrophage-inflammatory protein 2 [111,112,113]. Caution is, however, advised in over-interpreting such results since mice are not naturally colonized with *P. acnes* and their immune response to the bacterium and its products may not accurately reflect the human scenario. More interestingly, data on the potential role of CAMP factor 2 as an inflammatory molecule in human skin has come from a recent study using ex vivo skin explants, which demonstrated higher levels of the CAMP protein, as well as IL-8 and IL-1β, in samples from acne patients versus non-lesional skin [113]. Furthermore, incubation of the explants with a MAb to the CAMP factor attenuated both IL-8 and IL-1β providing some evidence for its inflammatory potential; this is an interesting observation in light of the observation that CAMP factor 1 also stimulates IL-8 production (see Section 3.7). The cytotoxic effects of CAMP factor 2 may partly reflect interactions of the protein with host cell sphingomyelinase [111].

As all phylogroups and strains of *P. acnes* produce CAMP factor 2, a critical consideration in relation to its selection as a therapeutic target is the effect vaccination may have on health-associated lineages of the bacterium, as well as those associated with acne. When produced by commensal bacteria, the term “virulence factor” may be better described as a “niche factor” or “host-adaptation factor” to reflect an overlapping role in host colonization and survival, as well as episodes of pathogenicity [27,115]. Other lines of evidence that support the idea that CAMP factor 2 is more than just a virulence factor are the observation that it may have been purged from other cutaneous propionibacteria where it was not essential for survival on the human host, alongside the demonstration that the CAMP factor 2 gene appears to be evolving under functional constraints similar to that seen with housekeeping genes [27]. Its presence in hair follicles and sebaceous glands of non-lesional skin is also consistent with a role in commensal existence, albeit at lower levels than found in acne lesions where some bacterial overgrowth may have occurred [113]; against this latter observation, Bek-Thompson et al. [98] did not detect any CAMP factor 2 in follicular casts from acne patients (see Section 3.8). Furthermore, knockout mutants for the CAMP factor show significantly reduced bacterial growth compared to wild type within an in vivo context (mouse ear), compared to growth in broth culture [113]. Further studies are clearly required to determine what effect CAMP factor 2 vaccination would have on health-associated lineages of *P. acnes* and other bacterial microbiota on the skin. It may be that local passive immunoprotection with therapeutic anti-CAMP factor antibodies is a safer and more appropriate approach than pre-pubertal immunization [112].

## 4. Conclusions

In conclusion, the last number of years has seen some very significant developments in our understanding of *P. acnes* biology and the capacity of this bacterium to cause human disease. The observation that certain lineages are acne-associated, while other appear to promote skin health, has breathed new life into the study of acne pathogenesis. The integration of data from phylogenetics, multi-omic, biochemical and host-microbe studies has also provided a more holistic understanding of the mechanisms driving the pathogenicity of certain strains, thus creating opportunities for the future development of new therapeutics. As lineages of *P. acnes* positively associated with skin health appear to exist, new antimicrobial treatments should ideally target acneic type IA_1_ lineages only, leaving beneficial strains intact and minimising any further dysbiosis.

## Figures and Tables

**Table 1:** Skin strain differences between acne and healthy subjects based on the study and data of Fitz-Gibbon et al. [66].

Ribotype	Type	MLST_8_ Clonal Complexes (CC)	N *	Percentage of Clones from Acne	Percentage of Clones from Controls	*p*-Value
RT1	IA_1_/IB	CC1; CC3; CC4; CC5	90	48%	52%	0.84
RT2	II	CC6; CC72	48	51%	49%	0.36
RT3	IA_2_	CC2	60	40%	60%	0.092
RT4	IA_1_	CC3	23	84%	16%	0.049
RT5	IA_1_/IC	CC1; CC3; CC107	15	99%	1%	0.0005
RT6	II	CC6	11	1%	99%	0.02
RT7	ND	ND	10	99%	1%	0.12
RT8	IA_1_	CC4	5	100%	0%	0.024
RT9	III	CC77	4	99%	1%	0.29
RT10	ND	ND	5	100%	0%	0.024

* N: Number of subjects.

**Table 2:** Key *P. acnes* proteins with putative effects on the host.

Putative Gene Function	KPA171202 ORF Number	Potential Effect on the Host
CAMP-factors	PPA0687, PPA1198, PPA1231, PPA1340, PPA2108	Haemolytic, cytotoxic
Haemolysins	PPA0565, PPA0938, PPA1396	Haemolytic
GehA lipases	PPA1796, PPA2105	Tissue damage, inflammation
Sialidases	PPA0684, PPA0685, PPA1560	Tissue damage
Hyaluronate lyase (HYL)	PPA0380	Tissue damage
Endoglycoceramidases	PPA0644, PPA2106	Tissue damage
Endo-ß-N-acetylglucosaminidase	PPA0990	Tissue damage
Dermatan sulphate adhesin (DsA1) (PTR) *	PPA2127	Colonization/adhesion/Fibrinogen-binding/inflammation
Dermatan sulphate adhesin (DsA2) (PTR) *	PPA2210	Colonization/adhesion/inflammation
PTRs *	PPA1715, PPA1879, PPA180, PPA1881, PPA1906, PPA2130, PPA2270	Colonization/adhesion/inflammation
GroEL	PPA0453, PPA1772, PPA1773	Inflammation
DnaK	PPA1098, PPA2040	Inflammation
DnaJ	PPA0916, PPA2038	Inflammation

* PTRs: Proline-Threonine Repeats likely involved in cell-cell and/or cell-matrix interactions.
